# P06 - Pulmonary functions are affected during pollen season in children with allergic rhinitis

**DOI:** 10.1186/2045-7022-4-S1-P61

**Published:** 2014-02-28

**Authors:** Süleyman Tolga Yavuz, Mustafa Güleç, Osman Şener

**Affiliations:** 1 Department of Pediatric Allergy, Gulhane Military School of Medicine, Ankara, Turkey; 2 Department of Adult Allergy and Clinical Immunology, Gulhane Military School of Medicine, Ankara, Turkey

## Background

Along with the nasal symptoms**,** asthma-like symptoms, particularly cough, may also be present in children with pollen-induced allergic rhinitis during pollen season. The aim of this study was to evaluate whether patients with pollen-induced allergic rhinitis without a physician diagnosed asthma might, nevertheless, have airways obstruction both in and out of the pollen season.

## Method

Patients with allergic rhinitis and grass pollen sensitization were evaluated both during and outside the pollen season. Allergic rhinitis diagnosis and severity grading were made according to ARIA guidelines. Skin prick tests, blood eosinophil counts and total IgE level measurements and pulmonary function tests (during and outside of pollen season) were performed in all subjects.

## Results

Thirty patients (17 male, 56.7%) with a median age of 11.5 (8.7-13.6) years were included. 12 patients (40%) had mild allergic rhinitis, whereas 18 (60%) had moderate-persistent allergic rhinitis interfering with daily activities. There was no significant difference at spirometric parameters including forced vital capacity (FVC), forced expiratory volume at 1 second (FEV_1_) and FEV1/FVC ratio, which obtained in and out of pollen season. However, the forced expiratory flow between the 25 and 75% of the vital capacity (FEF_25-75_) (100 (91-96) vs. 91 (83-104)) and peak expiratory flow (PEF) (92 (83-97) vs. 82 (76-94)) values were significantly lower in pollen season when compared with the values obtained out of pollen season.

## Conclusion

Pulmonary functions may be affected in children with pollen-induced allergic rhinitis during pollen season even if they have no history and symptoms of bronchial asthma.

